# Echicetin Coated Polystyrene Beads: A Novel Tool to Investigate GPIb-Specific Platelet Activation and Aggregation

**DOI:** 10.1371/journal.pone.0093569

**Published:** 2014-04-04

**Authors:** Alexey Navdaev, Hariharan Subramanian, Alexey Petunin, Kenneth J. Clemetson, Stepan Gambaryan, Ulrich Walter

**Affiliations:** 1 Institute of Clinical Biochemistry and Pathobiochemistry, University of Wuerzburg, Wuerzburg, Germany; 2 Institute of Biophysics, Siberian Branch of the Russian Academy of Sciences, Krasnoyarsk, Russia; 3 Theodor Kocher Institute, University of Berne, Berne, Switzerland; 4 Sechenov Institute of Evolutionary Physiology and Biochemistry, Russian Academy of Sciences, St. Petersburg, Russia; 5 Center for Thrombosis and Hemostasis (CTH), University Medical Center Mainz, Mainz, Germany; King’s College London School of Medicine, United Kingdom

## Abstract

von Willebrand factor/ristocetin (vWF/R) induces GPIb-dependent platelet agglutination and activation of αIIbβ3 integrin, which also binds vWF. These conditions make it difficult to investigate GPIb-specific signaling pathways in washed platelets. Here, we investigated the specific mechanisms of GPIb signaling using echicetin-coated polystyrene beads, which specifically activate GPIb. We compared platelet activation induced by echicetin beads to vWF/R. Human platelets were stimulated with polystyrene beads coated with increasing amounts of echicetin and platelet activation by echicetin beads was then investigated to reveal GPIb specific signaling. Echicetin beads induced αIIbβ3-dependent aggregation of washed platelets, while under the same conditions vWF/R treatment led only to αIIbβ3-independent platelet agglutination. The average distance between the echicetin molecules on the polystyrene beads must be less than 7 nm for full platelet activation, while the total amount of echicetin used for activation is not critical. Echicetin beads induced strong phosphorylation of several proteins including p38, ERK and PKB. Synergistic signaling via P2Y_12_ and thromboxane receptor through secreted ADP and TxA_2_, respectively, were important for echicetin bead triggered platelet activation. Activation of PKG by the NO/sGC/cGMP pathway inhibited echicetin bead-induced platelet aggregation. Echicetin-coated beads are powerful and reliable tools to study signaling in human platelets activated solely via GPIb and GPIb-triggered pathways.

## Introduction

In vivo, von Willebrand Factor (vWF) interacts with platelet glycoprotein Ib complex (GPIb) under high shear and triggers platelet activation, which leads to ADP and TxA_2_ release and hence αIIbβ3 activation and platelet aggregation. Under normal conditions, vWF does not interact with circulating platelets [1]. Following injury under high shear conditions the globular molecule of vWF adheres to subendothelial collagen and is stretched to expose the A1 domains, the binding domain for GPIb [2]. vWF is a large multimer containing many A1 domains, which can bind GPIb molecules on the platelet surface [3]. The clustering of GPIb receptors linked to the submembranous cytoskeleton triggers platelet activation [4], [5]. However, the number of A1 domains interacting with GPIb on a platelet surface as well as the distance between neighbouring A1 domains required for GPIb clustering and platelet activation was not previously determined.

The antibiotic ristocetin binds to the C-terminal part of the A1-domain of vWF as well as to GPIb, inducing the interaction between vWF and GPIb on platelets [6]. However, vWF/R treatment strongly agglutinates stirred, washed platelets [7]. Agglutination by vWF/R is passive and does not cause intracellular signaling. It takes place even with formaldehyde-fixed platelets (often used as a clinical test). The C1 domain of vWF can also interact with αIIbβ3 integrin on the platelet surface to induce an intracellular signal independently from GPIb [8], [9]. All these events caused by vWF/R treatment obscure the analysis of GPIb receptor signaling. A surface coated with recombinant dimeric A1 domain was used earlier to study GPIb specific signaling [10]. However, dimeric A1 domain cannot be used to activate platelets in suspension via their vWF receptor without cross linking the A1 domains. Therefore, we developed a new model for the study of GPIb specific signaling in human platelets based on echicetin-coated polystyrene beads (EP).

Echicetin, a snake venom C-type lectin, specifically binds to GPIb, but does not induce activation of washed platelets. Moreover, binding of echicetin to GPIb completely blocks vWF/R induced platelet agglutination [11]. However, echicetin can be cross-linked by plasma IgMκ to induce platelet agglutination and weak aggregation [12]. A maximum of 10 echicetin molecules can bind to one IgMκ molecule and this complex may not be sufficient to cluster GPIb molecules to the extent required to activate αIIbβ3 and induce full aggregation. Therefore, we used echicetin coated polystyrene beads with a determined number of GPIb-binding sites and a known average distance between neighboring echicetin molecules, and investigated platelet agglutination/aggregation. We showed that our model can be used to study signaling mechanisms in platelets specifically activated via GPIb and that for maximum platelet activation and aggregation the GPIb ligands should be spaced closer than 7 nm.

## Materials and Methods

### Ethics Statement

Blood was obtained from healthy volunteers, who gave written informed consent according to our institutional guidelines and the Declaration of Helsinki. Our studies with human platelets and the consent procedure were approved and reconfirmed (September 24, 2008) by the local ethics committee of the University of Wuerzburg (Studies No. 67/92 and 114/04).

### Materials

*Echis carinatus sochureki* venom was from Latoxan (Valence, France). Ristocetin was from Biopool (Wicklow, Ireland). vWF (Haemate HS 1000, vWF activity 2200 IE) was from CSL Behring (Marburg, Germany). Polystyrene beads (0.46 μm, aqueous suspension, 10% solids) and phospho–p38 (Thr^180^/Tyr^182^) MAPK antibody were from Sigma (Taufkirchen, Germany). R-phycoerythrin (RPE)-conjugated anti-CD62P antibody was from DAKO (Glostrup, Denmark). Anti-phosphotyrosine antibody (4 G10) was from Millipore (Schwalbach, Germany). Phospho-VASP Ser^239^, and phospho-ERK (Thr^201^/Tyr^204^) antibodies were from Nanotools (Teningen, Germany). Antibodies against phospho-PKB (Thr^308^), VASP, and p38 were from Cell Signaling (Frankfurt, Germany).

### Preparation of Echicetin-coated Polystyrene Beads

Echicetin was purified as previously described [12]. Polystyrene beads were washed and coated with echicetin in PBS overnight followed by washing and blocking with BSA. To obtain different densities, the beads were coated with 0.3 (EP0.3), 0.2 (EP0.2), 0.1 (EP0.1) and 0.05 (EP0.05) mg/ml echicetin.

### Determination of the Amount of Echicetin, and the Distance between the Echicetin Molecules on the Beads

Echicetin was labeled with I^125^ using Pierce Iodination Reagent (Iodogen) with a standard protocol (Thermo Scientific - Pierce, Rockford, USA). Unreacted I^125^ was removed from the labeled protein by gel-filtration on Sephadex G10. Labeled echicetin (10 μg) was mixed with 1 mg of unlabeled echicetin. This mixture was used as a stock to prepare 0.3 (EP0.3), 0.2 (EP0.2), 0.1 (EP0.1) and 0.05 mg/ml (EP0.05) echicetin solutions for adsorption on polystyrene beads using the same protocol as with unlabeled protein. The total radioactivity of the samples was measured before adsorption and the residual radioactivity on polystyrene beads was measured after adsorption using a gamma-counter. The amount of adsorbed echicetin was calculated from the ratio between the total sample radioactivity and the residual radioactivity on the polystyrene beads. We found that 28.2, 23.7, 13.2 and 7.5 μg of echicetin (30.6 kDa) bound to EP0.3, EP0.2, EP0.1 and EP0.05, respectively. Absorbed echicetin (A) corresponds to 5.7×10^14^, 4.8×10^14^, 2.7×10^14^, and 1.5×10^14^ molecules. To find the average distance between two neighboring echicetin molecules absorbed on polystyrene beads the total area of all beads in the suspension was calculated as follows.
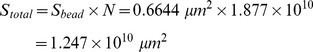
where S_bead_ is the surface area of a single bead (0.6644 μm^2^ for a bead with d = 0.46 μm) and N is the total number of beads in the sample, calculated using an equation supplied by Sigma and determined to be 1.877×10^10^.

Assuming that echicetin is evenly distributed on the polystyrene surface, the mean area on the bead occupied by each echicetin molecule was calculated by the equation: 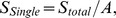 where S_single_ is the area containing a single molecule, S_total_ is the total area of polystyrene beads covered by echicetin and A is the number of adsorbed echicetin molecules. For EP0.3, EP0.2, EP0.1 and EP0.05, S_single_ was found to be 21.8, 25.7, 46.2 and 81.2 nm^2^ respectively. The distance between two neighboring molecules (D) was calculated as 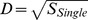 and found to be 4.7, 5.1, 6.8 and 9.0 nm for EP0.3, EP0.2, EP0.1 and EP0.05, respectively. Echicetin molecules per bead were calculated as n = A/N giving 30500, 25800, 14300 and 8170 for EP0.3, EP0.2, EP0.1 and EP0.05, respectively.

### Platelet Aggregation

Human platelets were prepared as previously described [13]. Platelet aggregation was measured using an Apact4004 (LabiTec, Ahrensburg, Germany) aggregometer. Washed platelets (3×10^8^/ml) were treated with echicetin beads (1% w/w suspension) or vWF/R under continuous stirring at 1000 rpm and 37°C.

### Flow Cytometry

Platelets activated with echicetin beads were taken directly from the aggregation cuvette and fixed with 1% formaldehyde for 10 min. After centrifugation, platelets were resuspended in PBS/0.5% BSA, and incubated with RPE-conjugated anti-CD62P antibody. FACS analysis was performed on a Becton Dickinson FACScalibur using CELLQuest software, version 3.1f (Becton Dickinson, Heidelberg, Germany).

### Platelet Adhesion and Spreading

Cover slips were coated with various concentrations of echicetin. Platelet adhesion and spreading on echicetin coated surfaces were performed as described before [14]. Adhered platelets were studied under the microscope (Zeiss, Axiovert 200) at 1000× magnification. Platelet images were captured using a camera (Diagnostic Instruments, SpotPursuit 23) and VisiView software.

### Western Blot Analysis

For Western blot analysis, 2× Laemmli buffer was directly added to washed platelets stimulated with echicetin beads or vWF/R. Platelet proteins were separated by SDS-PAGE, transferred to nitrocellulose membranes, and the membranes were incubated with appropriate primary antibodies. Goat anti-rabbit or anti-mouse IgG conjugated with horseradish peroxidase were used as secondary antibodies, followed by ECL detection.

### Data Analysis

All experiments were performed at least three times and the data are expressed as mean ± SEM. Differences between groups were analyzed by ANOVA followed by Bonferroni’s test and Student t-test was used when appropriate. P<0.05 was considered statistically significant.

## Results

### Echicetin Beads Induce αIIbβ3-dependent Platelet Aggregation

**Figure 1:**
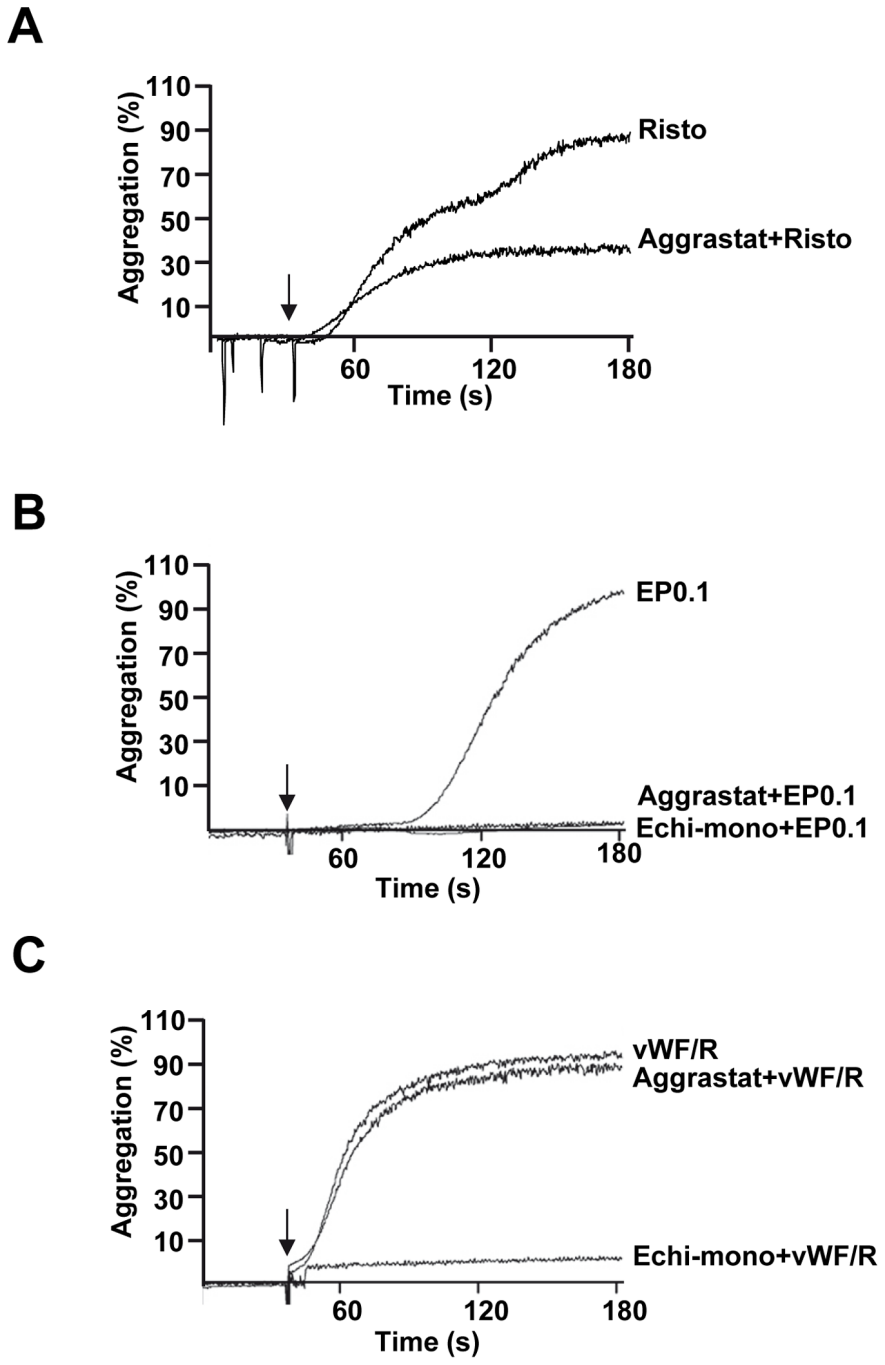
Echicetin coated polystyrene beads induce αIIbβ3–dependent aggregation in washed platelets. Representative aggregation curves of platelet rich plasma stimulated with ristocetin (A), and washed human platelets stimulated with EP0.1 in the absence of vWF or fibrinogen (B) or vWF-15 μg/ml and Ristocetin-1 mg/ml (C). Whenever indicated, platelets were preincubated with echicetin-monomer (25 μg/ml, 3 min) or aggrastat (1.25 μg/ml, 1 min). Shown are results of three independent experiments.

Elucidation of intracellular signaling mechanisms induced by vWF/R in washed platelet suspension is limited by strong platelet agglutination. In PRP, addition of ristocetin induces two waves of platelet activation; first one is agglutination, and the second αIIbβ3-dependent aggregation (Fig. 1A). Therefore, to examine GPIb specific signaling in washed platelets suspension, we introduced a new model of echicetin coated polystyrene beads (EP). Echicetin was shown to bind specifically to GPIb and to block platelet interactions with vWF [11], [12]. EP, after a short lag-phase, induced strong platelet aggregation, which was completely blocked by preincubation with αIIbβ3 inhibitor aggrastat, or echicetin monomer (Fig. 1B). In contrast to PRP, in washed platelet suspension, agglutination of platelets by vWF/R was inhibited only by monomeric echicetin, but not by aggrastat (Fig. 1C).

**Figure 2:**
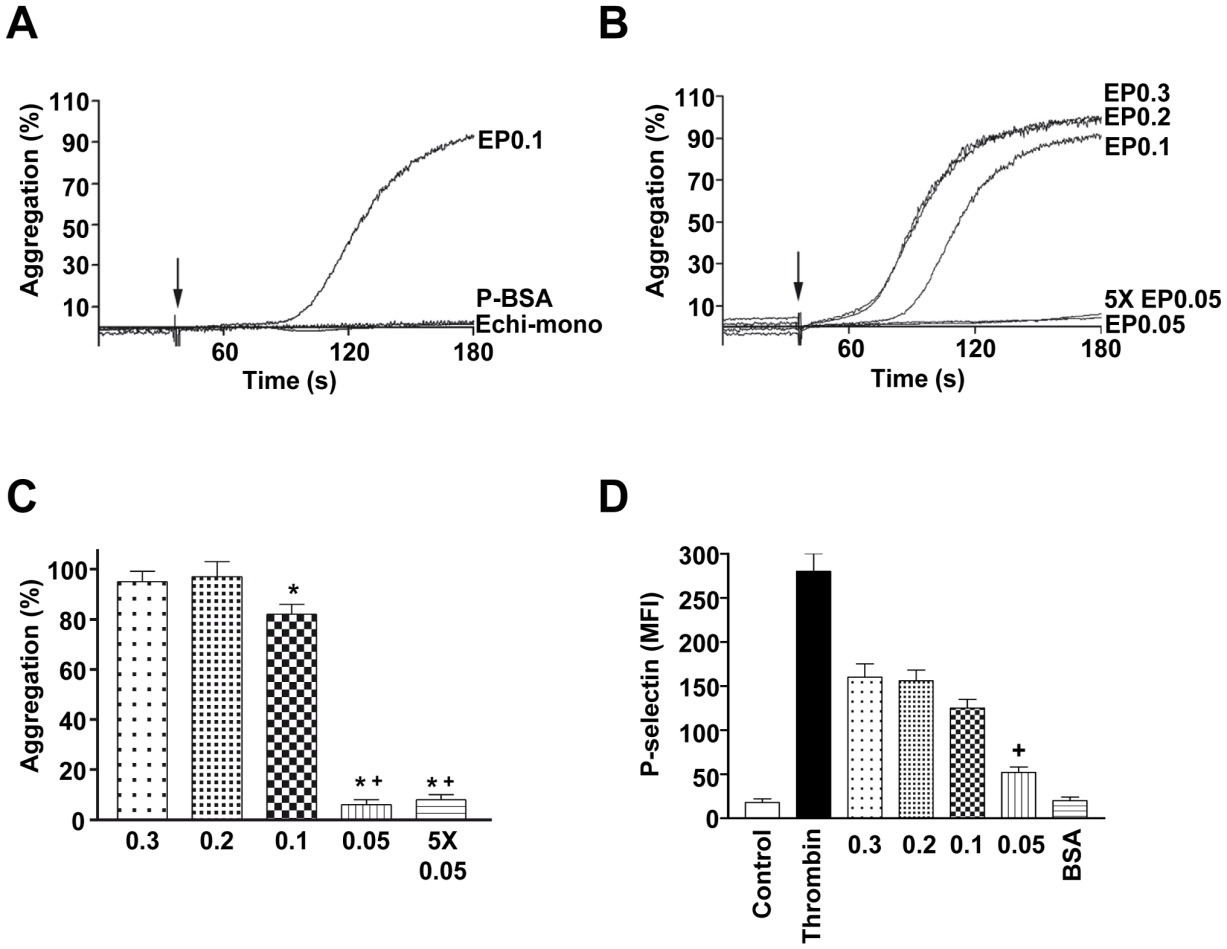
The distance between echicetin molecules is critical for inducing platelet activation and aggregation. Washed human platelets were stimulated with EP under stirring conditions (1000 rpm). BSA coated polystyrene beads were used as negative control. Platelet aggregation stimulated by EP0.3 was considered as 100%. A. Representative aggregation curves of platelets stimulated with EP0.1, BSA-coated polystyrene beads, or echicetin-monomer are shown. B. Representative aggregation curves of platelets stimulated with EP0.3, EP0.2, EP0.1, EP0.05 or 5 times concentration of EP0.05 are shown. C. The graph shows the corresponding average values of aggregation from B. D. P-selectin surface expression on platelets was measured as Mean Fluorescence Intensity (MFI) using RPE-conjugated P-selectin antibody in flow cytometry. Thrombin (0.05 U/ml) and BSA-coated beads (BSA) were used as positive and negative controls, respectively. Results are mean±SD from 3 independent experiments. * p<0.05 versus EP0.3, and^+^p<0.05 versus EP0.1.

Neither monomeric echicetin nor BSA coated beads (used as controls) induce platelet aggregation or agglutination (Fig. 2A). To elucidate whether the density, or the distance between neighboring molecules of absorbed echicetin influences the ability of EP to induce platelet aggregation, polystyrene beads were coated with a defined concentration of echicetin based on the data obtained with absorption of I^125^ labeled echicetin on polystyrene beads (see methods). First, we established the range of echicetin concentrations (between 1 and 0.01 mg/ml) for coating the beads and found that maximum aggregation was induced by incubation with 0.3 mg/ml, whereas 0.05 mg/ml did not induce any significant platelet aggregation. According to our calculations (see methods) the number of echicetin molecules per bead should be 30500, 25800, 14300 and 8170 in EP0.3, EP0.2, EP0.1, and EP0.05, respectively and the average distance between the adsorbed echicetin molecules is 4.7, 5.1, 6.8 and 9.0 nm. Correspondingly, the total number of echicetin molecules added to platelet samples were 5.7×10^14^, 4.8×10^14^, 2.7×10^14^, and 1.5×10^14^ for EP0.3, EP0.2, EP0.1, and EP0.05, respectively. There are about 40000 molecules of GPIb per platelet [15] and the total number of GPIb molecules in 4.6×10^7^ platelets used in aggregation experiments were 1.84×10^12^ approximately, which is comparable with the total number of echicetin molecules in PE0.05.

The maximum aggregation, 94.6±5.6% and 94.9±10.8% was obtained when platelets were activated by EP0.3 and EP0.2, respectively. Slightly less aggregation (86.1±8.2%) was observed with EP0.1 (Fig. 2B, 2C). However, EP0.05 did not induce aggregation (3.2±1.3%), indicating that the amount, and/or the distance between echicetin molecules in the beads were not sufficient for GPIb mediated platelet aggregation. In order to clarify this, we added 5 times more EP0.05 (7.5×10^14^ echicetin molecules per sample) and still found no significant changes in platelet aggregation (Fig. 2C). These data suggest that the distance between the echicetin molecules is critical, but not the total number of echicetin molecules on the beads used for stimulation. The average distance between echicetin molecules should be less than 7 nm for GPIb mediated platelet aggregation.

### Echicetin-coated Beads Induce P-selectin Surface Expression in Human Platelets

Platelet activation induced by echicetin beads was determined by P-selectin expression on the activated platelets. Though thrombin and echicetin beads induce full platelet aggregation, P-selectin expression induced by echicetin beads was significantly less than in thrombin-stimulated platelets. There were no significant differences between P-selectin expression levels induced by EP0.3 and EP0.2. P-selectin expression was slightly lower with EP0.1 and strongly decreased in the case of EP0.05 (Fig. 2D). These data show that echicetin beads induce alpha-granule secretion, but less than strong agonists like thrombin.

### Platelet Adhesion and Spreading on Echicetin-coated Surface

**Figure 3:**
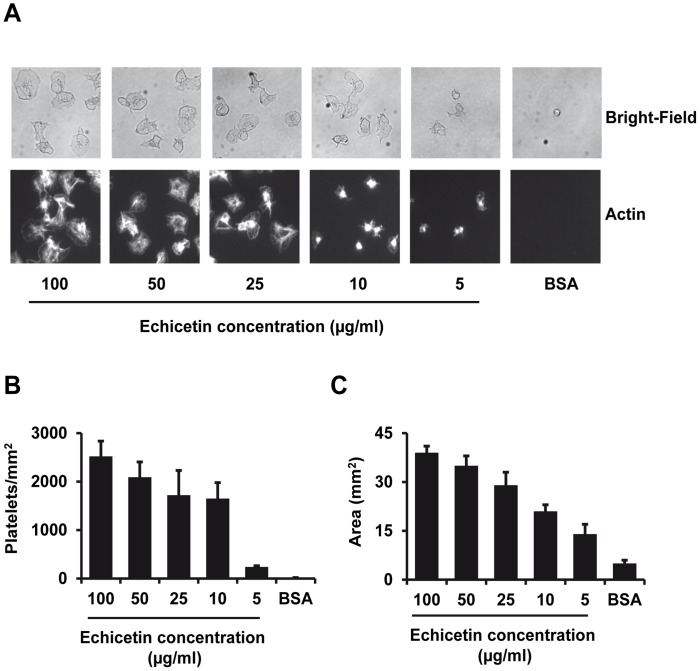
Platelet adhesion and spreading on echicetin-coated surface. Cover slips were coated with various amounts of echicetin or BSA (control). Washed human platelets (1×10^7^/ml) were incubated on the coated cover slips for 15 min. Following incubation, cover slips were washed to remove unbound platelets and the adherent platelets were fixed with 1% formaldehyde. The fixed platelets were permeabilized with Triton X-100 and stained with phalloidin-Oregon Green (actin). The stained platelets were analyzed under the microscope (Zeiss, Axiovert 200) at 1000× magnification. The images were captured using a camera (Diagnostic Instruments, SpotPursuit 23) and VisiView software. A. Representative images of the bright-field (upper panel) and actin staining (lower panel) of the cover slips were shown for each sample. B. The number of adhered platelets was counted in 10 independent cover slips for each sample. C. Surface area of adhered and spread platelets was measured using VisiView software in 10 independent cover slips for each sample. Results are mean ± SEM from 3 independent experiments.

To identify whether GPIb-mediated platelet activation induces platelet adhesion and spreading, washed human platelets were incubated on cover slips coated with increasing concentrations of echicetin. We found typical platelet adhesion and spreading on echicetin coated surfaces and the number of adherent platelets increased with the density of echicetin coated on the surface (Figure 3A, B). The area covered by spread platelets was also dependent on the concentration of the echicetin coated on the surface (Figure 3C). These results indicated that GPIb-mediated platelet activation by echicetin induces platelet adhesion and spreading.

### Echicetin Beads Induce Tyrosine Phosphorylation in Platelets

**Figure 4:**
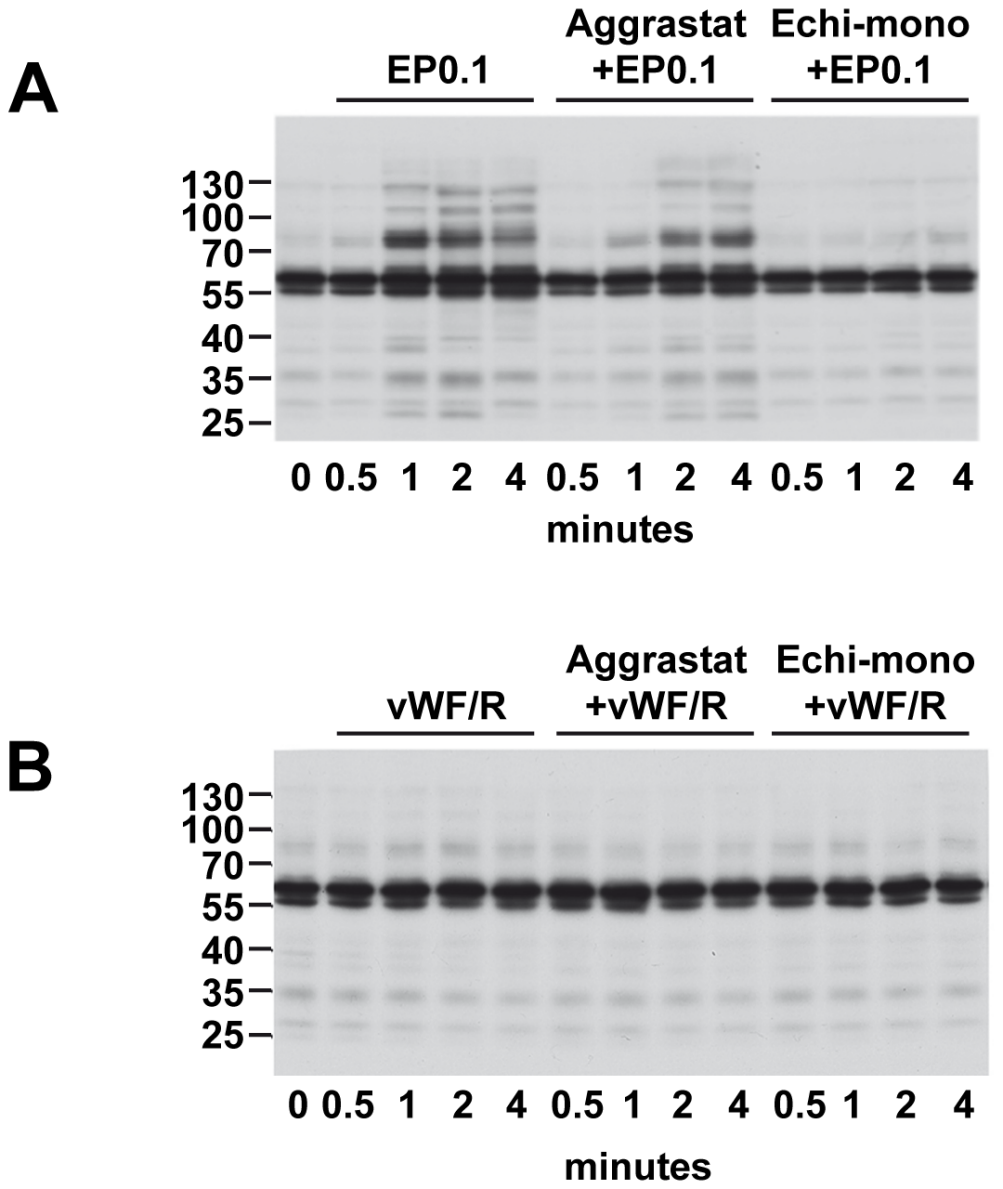
Echicetin beads but not vWF/R induces protein tyrosine phosphorylation. Washed human platelets were stimulated with EP0.1 (A) or vWF/R (B) after preincubation with aggrastat (1.25 μg/ml, 1 min) or echicetin monomer (25 μg/ml, 3 min). Protein tyrosine phosphorylation was analyzed by Western blot. Shown are representative blots of three independent experiments.

Global protein tyrosine phosphorylation is a reliable marker of activation dependent intracellular signaling. Therefore, we examined changes in protein tyrosine phosphorylation in platelets activated by EP0.1. Strongly increased tyrosine phosphorylation of several proteins was observed in platelets stimulated by EP0.1, which was partially inhibited by aggrastat (Fig. 4A). This indicates that GPIb-induced tyrosine phosphorylation is partly mediated by outside-in integrin signaling most likely via proteins released from α-granules such as fibrinogen. However, preincubation with monomeric echicetin completely abolished the increase in tyrosine phosphorylation induced by EP0.1 (Fig. 4A). In contrast, under our experimental conditions, activation of platelets with vWF/R did not trigger any tyrosine phosphorylation (Fig. 4B), which directly corresponds to the platelet agglutination data (Fig. 1C).

### Platelet Aggregation Induced by Echicetin Beads is Mediated by Src-kinases and SYK

**Figure 5:**
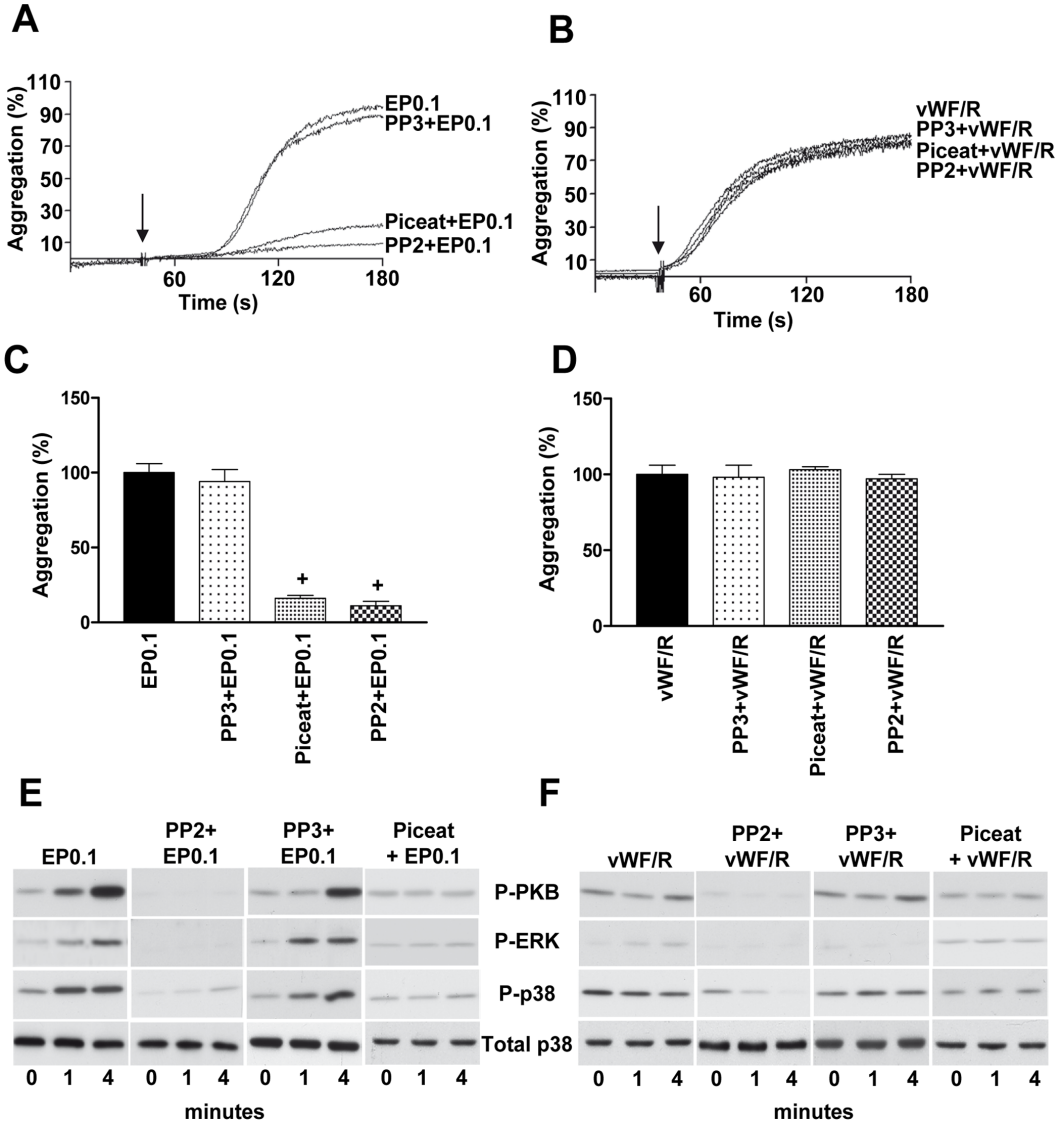
Src-kinases and SYK are downstream effectors of echicetin beads induced platelet activation. Washed human platelets were preincubated with Src-kinases inhibitor (PP2, 10 μM), an inactive analog (PP3, 10 μM) or Syk inhibitor (piceatannol, 10 μM) before stimulating with EP0.1 or vWF/R under stirring conditions. Representative aggregation curves of platelets stimulated with EP0.1 (A) or vWF/R (B) are shown. C, D. The graphs shows corresponding average values of aggregation from A and B. Platelets stimulated with EP0.1 (E) or vWF/R (F) were lysed at the indicated time and the phosphorylation of PKB, ERK and p38 was analyzed by Western blot. Total p38 served as loading control. Shown are representative blots of three independent experiments.^+^p<0.05 versus EP0.1.

Platelets preincubated with Src-kinase inhibitor (PP2) did not form aggregates, when stimulated with EP. PP3, an inactive analog of PP2 did not affect EP-induced platelet aggregation (Fig. 5A, C). In contrast, PP2 had no effect on vWF/R-induced platelet agglutination (Fig. 5B, D). GPIb-mediated platelet signaling was also tested using piceatannol, a SYK specific inhibitor. Piceatannol strongly inhibits aggregation induced by echicetin beads (Fig. 5A, C) and had no effect on vWF/R-induced agglutination (Fig. 5B, D). MAP kinases (p38, ERK) and PKB play essential role in vWF-stimulated platelet activation [16], [17], [18]. Therefore, we examined the phosphorylation of p38, ERK and PKB in EP-activated platelets. EP-induced phosphorylation of p38, ERK and PKB were completely inhibited in platelets by pre-incubation with PP2 and piceatannol (Fig. 5E). In contrast, in vWF/R treated platelets, PKB and MAP kinases were not activated and Src kinase and Syk inhibitors inhibited only basal phosphorylation of these kinases (Fig. 5F).

### ADP and TXA_2_ Signaling are Important for Echicetin Bead Induced Platelet Activation and Aggregation

**Figure 6:**
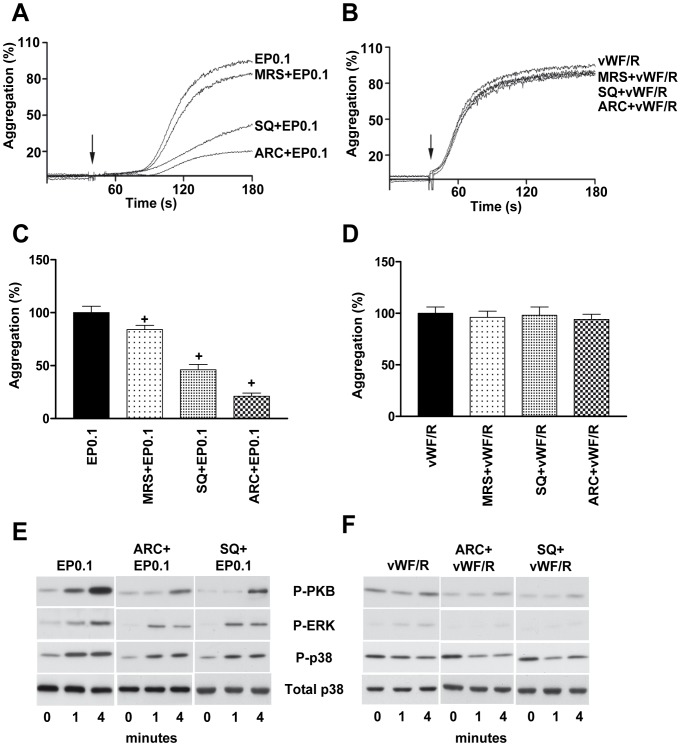
ADP and TxA_2_ play significant roles in echicetin bead induced platelet aggregation. Washed human platelets were preincubated with P2Y_12_ antagonist (AR-C69931; 0.1 μM, 5 min), P2Y_1_ antagonist (MRS2179; 1 μM, 5 min) or TxA_2_ receptor antagonist (SQ-29548; 1 μM, 5 min) before EP0.1 or vWF/R stimulation. Representative aggregation curves of platelets stimulated with EP0.1 (A) or vWF/R (B) are shown. C, D. The graphs shows corresponding average values of aggregation from A and B. Platelets stimulated with EP0.1 (**E**) or vWF/R (**F**) were lysed on indicated time and the phosphorylation of PKB, ERK and p38 was analyzed by Western blot. Representative blots of three independent experiments are shown.^+^p<0.05 versus EP0.1.

Secreted ADP and TXA_2_ play significant roles in shear-induced GPIb mediated platelet aggregation [1], [19]. Mouse platelets in suspension stimulated with botrocetin/vWF also required ADP and TXA_2_ for aggregation [20], [21]. To assess the role of ADP and TxA_2_ signaling in EP-induced platelet aggregation, platelets were pre-incubated with antagonists of P2Y_12_ (AR-C69931), P2Y_1_ (MRS-2179) or TxA_2_ receptor (SQ-29548). AR-C69931 potently inhibits EP-induced platelet aggregation, whereas MRS-2179 had only a minimal effect. SQ-29548 strongly reduced EP-induced platelet aggregation (Fig. 6A, C). However, simultaneous inhibition of ADP and TXA_2_ receptors did not significantly differ from the effect of AR-C69931 alone (data not shown) indicating that other intracellular mechanisms are involved in EP-induced platelet aggregation. Pre-treatment with AR-C69931 and SQ-29548 inhibits phosphorylation of PKB, ERK and p38 (Fig. 6E) in EP-stimulated platelets. In contrast, neither ADP nor TxA_2_ receptor antagonists affect vWF-induced platelet agglutination (Fig. 6B, D) and phosphorylation of PKB, ERK and p38 (Fig. 6F).

### NO-dependent Activation of Soluble Guanylate Cyclase Inhibits Echicetin Beads-induced Platelet Aggregation

**Figure 7:**
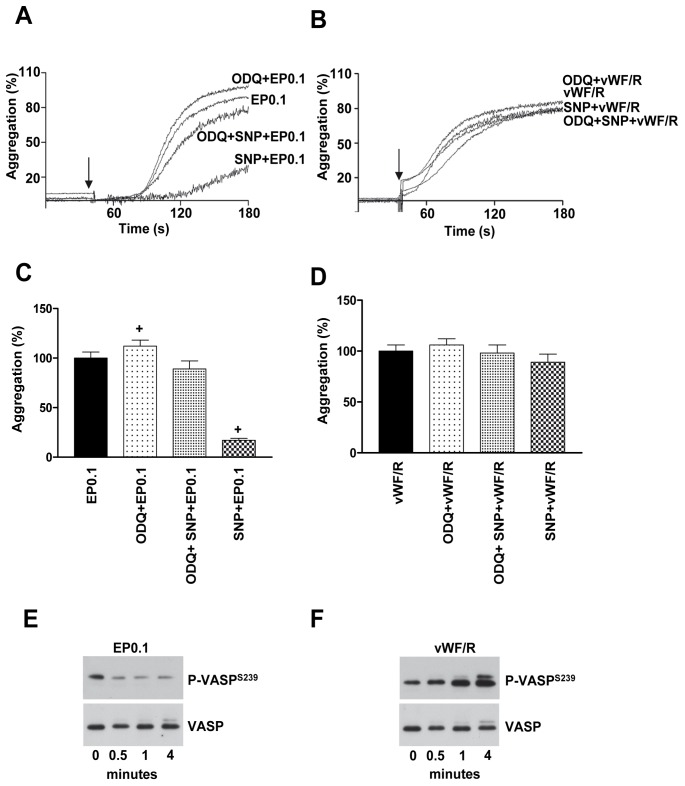
Activation of sGC inhibits platelet aggregation induced by echicetin beads. Washed human platelets were preincubated with NO donor (SNP; 10 μM, 2 min) and/or sGC inhibitor (ODQ; 50 μM, 5 min) before EP0.1 or vWF/R stimulation. Representative aggregation curves of platelets stimulated with EP0.1 (A) or vWF/R (B) are shown. C, D. The graphs show corresponding average values of aggregation from A and B. Platelets stimulated with EP0.1 (E) or vWF/R (F) were lysed and the phosphorylation VASP was analyzed by Western blot. Total VASP served as loading control. Representative blots of three independent experiments are shown.^+^p<0.05 versus EP0.1.

Previously, we [22], [23] and others [24], [25] found that vWF/R treatment of platelets activates the sGC/cGMP/PKG pathway and induces VASP phosphorylation. To evaluate whether in this case VASP phosphorylation is mediated by direct activation via GPIb or by a complex reaction of vWF/R we used our established system (echicetin beads) to stimulate platelets. First, we found that PKG activation by sodium nitroprusside (NO donor) inhibits EP-induced platelet aggregation by 83±7% (Fig. 7A, C). Preincubation with sGC specific inhibitor (ODQ) significantly enhanced (12±3%) EP-induced platelet aggregation (Fig. 7A, C), but had no effect on vWF/R-induced agglutination (Fig. 7B, D). Second, we tested whether VASP phosphorylation in vWF/R treated platelets is mediated by GPIb signaling. We can confirm our previous finding [22] that vWF/R treatment induces strong PKG activation and subsequent VASP phosphorylation (Fig. 7F). However, specific activation of GPIb by EP0.1 does not induce any VASP phosphorylation (Fig. 7E). These data indicate that vWF/R induced PKG activation is not directly mediated by GPIb activation but requires additional signaling pathways induced by vWF/R.

## Discussion

vWF mediated signaling in platelets is complex and involves several intracellular mechanisms, which play an important role in the initiation of thrombus formation. The complexity of these mechanisms is related to the vWF molecule itself and its main platelet receptor, the GPIb/IX/V complex. vWF has several domains including the D domain (the binding site for FVIII and heparin), D’-D3 domain (possible binding site for P-selectin), A3 domain (the binding site for fibrillar collagen type I and III), C1 domain (the binding site for integrin α_IIb_β_3_), and A1 domain which binds GPIb [8]. In addition to vWF, other ligands like thrombospondin-1 [26] and α-thrombin [27] can bind to GPIb and induce intracellular signaling independently from vWF. Numerous approaches including genetically modified mouse models, various forms of von Willebrand disease, shear-induced thrombus formation, specific interactions with isolated A1 domain, etc. have been used for the characterization of vWF-induced signaling [28]. In models, where platelets are used in suspension additional modulators such as ristocetin or botrocetin need to be added to initiate vWF/GPIb mediated platelet response. However, in all these models identification of specific signaling pathways mediated solely by vWF/GPIb interactions is extremely difficult for the following reasons: Apart from GPIb, vWF can bind to other receptors on the platelet surface and initiate complex signaling events; vWF/R or vWF/botrocetin, under stirring conditions (which are important to initiate aggregation of platelet suspensions), induce strong platelet agglutination, which can influence intracellular signaling mediated by vWF/GPIb interaction. The degree of agglutination probably depends on the source of vWF and the experimental conditions. Strong platelet agglutination (which is independent of integrin α_IIb_β_3_) induced by vWF/R treatment has been described in several papers [7], [29], [30], [31], [32]. In our study, we used Haemate HS (vWF activity 2200IE), which is clinically used as vWF substitute in vWF deficiencies (von Willebrand diseases). We observed that vWF/R treatment induces only strong platelet agglutination, which was not affected by aggrastat (Fig. 1C), inhibitors of Src kinase and SYK (Fig. 5), ADP and TXA_2_ receptors (Fig. 6).

The main aim of our study was to develop a GPIb-specific agonist to investigate the GPIb-mediated signaling events in platelets without interference from other receptor pathways. Echicetin, a C-type lectin from snake venom, seems to be applicable for investigations addressing GPIb specific signaling in washed platelet suspension. Previously, we had shown that a maximum of 10 echicetin molecules cross-linked with IgMκ can induce αIIbβ3-independent platelet agglutination [12]. The distance between the echicetin molecules coupled to IgMκ is sufficient to cluster few GPIb molecules that lead to agglutination. However, this complex did not allow us to find out the distance between echicetin molecules required to induce αIIbβ3 integrin-dependent platelet aggregation. Therefore, we developed a novel tool to investigate GPIb-specific activation of αIIbβ3 integrin-dependent platelet aggregation. With this tool, we identified that the distance between the echicetin molecules (<7 nm), but not the amount of echicetin is critical to induce GPIb clustering and GPIb-mediated αIIbβ3 integrin activation (Fig. 2). Also, platelet adhesion and spreading depend on the density of echicetin on the surface used to activate platelets (Fig. 3). These data strongly support the hypothesis that clustering of GPIb molecules is an important factor for the initiation of GPIb-mediated intracellular signaling.

We then compared the aggregation/agglutination induced by EP and vWF/R and found, consistent with published data [24], [33], that vWF/R induces strong agglutination in washed platelet suspension (Fig. 1C), without significant activation of tyrosine kinases (Fig. 4B). In contrast to washed platelets, in PRP addition of ristocetin induced the well characterized two waves of platelet activation; first agglutination, which is insensitive to aggrastat, and second, aggregation inhibited by aggrastat (Fig. 1A). Whereas EP, under the same experimental conditions, induces strong global tyrosine phosphorylation, which was only slightly decreased by αIIbβ3 inhibitor, indicating that most of the intracellular signal is mediated by activation of GPIb, but not αIIbβ3 outside-in signaling (Fig. 4A). It is important to note that adding echicetin monomer before stimulation by vWF/R or EP completely prevents platelet aggregation (Fig. 1B, C) and tyrosine phosphorylation (Fig. 4A). We also investigated several intracellular signaling pathways downstream of GPIb signaling, and consistent with others [16], [18], [24], [34], we found that Src kinases, SYK, PKB, p38 and ERK MAP kinases are strongly activated by GPIb signalling. However, none of these kinases were activated in vWF/R agglutinated platelets (Fig. 5). We found that P2Y_12_ and TxA_2_ signaling plays a synergistic role in EP-induced platelet activation and aggregation, in line with earlier findings [1], [19], [20], [21] (Fig. 6). In contrast with other publications [16], [21], [35], we did not find significant input from P2Y_1_ receptor in GPIb-induced platelet aggregation (Fig. 6). Our data clearly show that P2Y_1_ receptor has only a minor role in GPIb signaling. This finding does not imply that P2Y_1_ receptors are without a significant role in vWF-mediated platelet activation. Probably activation of the P2Y_1_ is connected with other pathways stimulated by vWF, but not by GPIb-mediated signaling.

We can clearly confirm previous findings [22], [23], [24], [25], [36] that vWF/R triggers the sGC/cGMP/PKG pathway (Fig. 7F). However, this pathway is not mediated by GPIb signaling, as we could show that EP did not increase phosphorylation of VASP (Fig. 7E), indicating that other, still unresolved mechanisms, connected with vWF/R are responsible for activation of the sGC/cGMP/PKG pathway.

In summary, we introduced a new agonist (echicetin coated polystyrene beads, EP) that activates platelets via clustering of GPIb without directly involving other receptors. This should allow the dissection of the complex mechanisms involved in vWF-induced platelet activation. We show that the distance (less than 7 nm) between GPIb ligands rather than their concentration is critical for platelet activation/aggregation. We propose that echicetin-coated beads can be a powerful and reliable tool to study specific GPIb-dependent signaling pathways in platelets.
